# Isolation and Characterization of Synovial Mesenchymal Stem Cell Derived from Hip Joints: A Comparative Analysis with a Matched Control Knee Group

**DOI:** 10.1155/2017/9312329

**Published:** 2017-01-01

**Authors:** Akihisa Hatakeyama, Soshi Uchida, Hajime Utsunomiya, Manabu Tsukamoto, Hirotaka Nakashima, Eiichiro Nakamura, Cecilia Pascual-Garrido, Ichiro Sekiya, Akinori Sakai

**Affiliations:** ^1^Department of Orthopaedic Surgery, University of Occupational and Environmental Health, Kitakyushu, Japan; ^2^Department of Orthopaedic Surgery, Wakamatsu Hospital, University of Occupational and Environmental Health, Kitakyushu, Japan; ^3^Department of Orthopaedic Surgery, Washington University School of Medicine, St. Louis, MO, USA; ^4^Department of Cartilage Regeneration, Tokyo Medical and Dental University, Tokyo, Japan

## Abstract

*Purpose*. To determine the characteristics of MSCs from hip and compare them to MSCs from knee.* Methods*. Synovial tissues were obtained from both the knee and the hip joints in 8 patients who underwent both hip and knee arthroscopies on the same day. MSCs were isolated from the knee and hip synovial samples. The capacities of MSCs were compared between both groups.* Results*. The number of cells per unit weight at passage 0 of synovium from the knee was significantly higher than that from the hip (*P* < 0.05). While it was possible to observe the growth of colonies in all the knee synovial fluid samples, it was impossible to culture cells from any of the hip samples. In adipogenesis experiments, the frequency of Oil Red-O-positive colonies and the gene expression of adipsin were significantly higher in knee than in hip. In osteogenesis experiments, the expression of COL1A1 and ALPP was significantly less in the knee synovium than in the hip synovium.* Conclusions*. MSCs obtained from hip joint have self-renewal and multilineage differentiation potentials. However, in matched donors, adipogenesis and osteogenesis potentials of MSCs from the knees are superior to those from the hips. Knee synovium may be a better source of MSC for potential use in hip diseases.

## 1. Introduction

An acetabular labral tear is one of the most common sources of hip pain and disability and one of the most frequent indications for hip arthroscopic surgeries. A concomitant labral tear with underlying femoroacetabular impingement (FAI) and/or dysplasia are risk factors for future cartilage damage and hip osteoarthritis [1].

Several standard procedures, including debridement and microfracture, have been routinely utilized to address chondral damage of the hip joint [2, 3]. In fact, there are studies that demonstrate favorable short to medium outcomes following microfracture treatment of localized cartilage damage in patients with FAI [4–6]. Other promising procedures such as osteochondral transplantation and autologous chondrocyte implantation (ACI) have been attempted to preserve cartilage for FAI patients [7]. However, studies on these newer procedures report consistently poor outcomes with high rates of conversion to total hip arthroplasty following hip arthroscopy for FAI patients with early to moderate osteoarthritis at the time of surgery [6, 8–10]. Thus, patients with severe cartilage damage are not good candidates for hip preservation surgery due to the high risk of failure.

Minimally invasive stem cell therapy may be a desirable strategy to treat cartilage lesions in the hip. Mesenchymal stem cells (MSCs) are incredibly unique candidates for use of cell therapy as they can be isolated from various tissue reservoirs such as bone marrow, adipose tissue, bone tissues, and synovial tissues [11, 12]. Previous in vitro studies demonstrated that MSCs can be isolated from synovial fluid in the knee joints [13]. Synovial MSCs derived from synovial tissues are superior to these other sources such as bone marrow and adipose tissues [14]. In addition, a recent in vivo study demonstrated that MSCs derived from synovial tissue have greater cartilage regenerating potential through comparing histological samples following knee injection in porcine animal models [15]. Sekiya et al. were able to translate this finding clinically and reported good outcomes following the use of synovial MSCs during arthroscopy for patients with cartilaginous knee injuries [16–18]. Much of the available literature is on MSCs derived from knee synovial tissues with a dearth of knowledge regarding MSCs derived from hip synovial tissues. Therefore, it is essential to evaluate the proliferation and differentiation potentials of MSCs derived from hip joints for future applications using MSCs therapy in the hip. This source of cells could potentially be beneficial for the treatment of hip disease such as focal chondral lesions and early OA.

The purpose of this study was (i) to isolate and define the characteristics of MSCs from synovial fluid and synovial tissues of the hip joint and (ii) to clarify whether there are any differences between synovial MSCs from the knee and the hip joint in the same donor. We hypothesize that MSCs derived from synovial fluid and synovial tissues of the hip joint can be isolated with the same proliferation and differentiation potential as those from the knee joint.

## 2. Materials and Methods

### 2.1. Patient Selection, Tissue Collection, and Isolation of Cells

Approval for the study was granted through the institutional review board. 423 hip arthroscopic surgeries were performed by a single surgeon between 2012 and 2014. Of 423 patients, 8 were eligible for this study as they suffered from both hip and knee intra-articular pathologies. Synovial samples were obtained from 2 male and 6 female patients (mean age ± SD; 47.9 ± 14.0 (range 25–64) years) during knee and hip arthroscopic surgeries. There were 3 premenopausal females and 3 postmenopausal females. There was no significant difference between the durations from onset of symptoms to date of surgery in these 8 patients. Furthermore, the preoperative osteoarthritis Kellgren-Lawrence grade on radiological classification between both joints for these patients was comparable (Table 1).

Arthroscopic surgeries for knee and hip pathologies in each eligible patient were performed on the same day. Human synovial fluid and synovium of the knee and the hip were obtained during arthroscopic surgery under general anesthesia. To minimize variation in sampling, specimens were harvested from the locations near the tear of meniscus in the knee and the torn labrum in the hip (Figure 2(a)). In Figure 2(a), when we harvested hip synovium, we released tissue junction between capsular ligament (iliofemoral ligament) and synovium and used a rongeur turning back on capsule to avoid the contamination of the capsule. Synovial fluid was obtained from the knee and the hip joints after injection of saline solution (50 mL). Synovium of the knee was obtained near the peripheral site at 5 mm distance from the injury of meniscus with a rongeur under arthroscopic observation. Synovium in the hip was also obtained from the peripheral area at 5 mm distance adjacent to the labral tear by using the same procedure. Arthroscopic findings of meniscus, labrum, and cartilage at the surgery and surgical procedure at both joints are shown in Table 1.

Synovial fluid was diluted with phosphate-buffered saline (PBS), filtered through 70 *μ*m nylon filter (Becton Dickinson, Franklin Lakes, NJ), and placed in six culture dishes (60 cm^2^ culture dish; Nalge Nunc International, Rochester, NY).

Both synovial samples were digested in 3 mg/mL collagenase from Type V (Sigma-Aldrich, St. Louis, Missouri) in *α*-modified Eagle's medium (*α*-MEM, Invitrogen, Carlsbad, CA) at 37°C. After 2 hours, the digested nucleated cells of synovium were plated at 10^3^, 10^4^, and 10^5^ cells per 60 cm^2^ culture dish (Nalge Nunc International, Rochester, NY), with 6 dishes created for each cell concentration. Cells were seeded in complete culture medium containing *α*-MEM and 20% fetal bovine serum (FBS). A high concentration of FBS was used to induce the highest number of colony-forming units after cell culture derived from hip synovial fluid among 6 lots of FBS. The cells were incubated at 37°C with 5% humidified CO_2_. After 3 to 4 days, the medium was changed to remove nonadherent cells. The adherent cells were then cultured for 14 days as passage 0 without refeeding. After 14 days, three dishes for each concentration were stained with 0.5% crystal violet. The optimal initial cell density was determined by (1) the resultant colony size being unaffected by contact inhibition and (2) a maximum in number of colonies being reached. We then harvested the cells plated at optimal densities from the remaining 3 dishes with Trypsin-EDTA (Invitrogen) and determined the number of cells at passage 0. The passage 0 cells were replated at 50 cells/cm^2^ in a 150 cm^2^ dish and cultured for 21 days. The cultured cells were cryopreserved by resuspension at a concentration of less than 10^6^ cells/mL in 10% fetal bovine serum. One-milliliter aliquots were slowly frozen to avoid cell damage and cryopreserved in liquid nitrogen (passage 1). To expand the cells, we thawed a frozen vial of the cells, plated the cells in 60 cm^2^ culture dishes, and incubated them for 4 days in a recovery plate. These cells (passage 2) were used for further analyses as previously described [19].

### 2.2. Estimation of the Yield Obtained

To investigate proliferation potential of synovial MSCs from the knee and the hip joints, we estimated the number of live cells at passage 0 gained from whole volume of each tissue and compared it to the final count of live cells from our cultures.

### 2.3. Histological Analysis

Synovial tissues obtained from the knee and the hip joints were fixed in a solution of 4% paraformaldehyde (PFA) with 0.1 mol/L phosphate buffer (PB) (pH 7.2), dehydrated through a graded series of alcohol, and embedded in paraffin. 5 *μ*m slice sections were prepared on microtome. These sections were stained with hematoxylin and eosin.

### 2.4. In Vitro Expandability and Cell Viability Assay

To examine the in vitro expandability of cells from each type of mesenchymal tissue, passage-1 cells were replated at 50 cells/cm^2^ in 150 cm^2^ culture dishes every 14 days until their growth potential was exhausted [14]. To determine the viabilities of cells in each passage, we analyzed quantitative evaluation on 3 donors with use of LIVE/DEAD Viability/Cytotoxicity Kit for mammalian cells (Invitrogen). In addition, a colorimetric assay for cell viability was conducted by plating passages 4 and 7 cells at 5000 cells in a 24-well multidish (Nalge Nunc International). These cells were cultured with 500 *μ*L of complete medium for 7 days. After incubation of the cells, 50 *μ*L of Cell Counting Kit-8 (highly water-soluble tetrazolium salt, WST-8, Dojindo, Kumamoto, Japan) was added to each well, and the dish was incubated for 1 hour. Cells were counted with a spectrophotometer multiplate reader 450 nm (SH1000, Corona Electric, Ibaraki, Japan). Cell viability was evaluated and expressed as a percentage based on the viable cell count of the synovium in knee (100%). At last, we analyzed the correlation between the fold increase and cell viability on each of the samples.

### 2.5. Differentiation Assays

#### 2.5.1. Adipogenesis in a Colony-Forming Assay

One hundred passage-2 cells were plated in 60 cm^2^ dishes and cultured in complete medium for 14 days. The medium was then switched to adipogenic medium *α*-MEM supplemented with 10% FBS, 10^−7^ M dexamethasone (Sigma-Aldrich), 0.5 mM isobutyl-1-methylxanthine (Sigma-Aldrich), and 50 *μ*M indomethacin (WAKO, Tokyo, Japan) for an additional 21 days. These cultured cells were fixed in 4% PFA and stained with fresh Oil Red-O (Sigma-Aldrich) solution. The number of Oil Red-O-positive colonies was then counted. The same adipogenic cultured cells were subsequently stained with crystal violet, and total cell colonies were counted.

#### 2.5.2. Osteogenesis in a Colony-Forming Assay

One hundred passage-2 cells were plated in 60 cm^2^ dishes and cultured for 14 days. The medium was then switched to osteogenic differentiation medium consisting of *α*con supplemented with 10% FBS, 10^−9^ M dexamethasone, 20 mM *β*-glycerophosphate (WAKO), and 50 *μ*g/mL ascorbate-2-phosphate for an additional 21 days. After 4% PFA fixation and positive colonies were counted, mineralized bone nodule formation was assessed by double labeling for mineral (von Kossa stain) and alkaline phosphate (ALP). The same osteogenic cultures were subsequently stained with crystal violet and the total cell colonies were counted. Colonies that were <2 mm in diameter and appeared yellow in color were discounted [14].

#### 2.5.3. In Vitro Chondrogenesis Using Pellet Culture Assay

250,000 passage-3 cells were placed in a 15 mL polypropylene tube (Becton Dickinson) and centrifuged at 450*g* for 10 min. The pellets were cultured in chondrogenesis medium (high-glucose Dulbecco's modified Eagle's medium (Invitrogen) supplemented with 500 ng/mL BMP-2, 10 ng/mL TGF *β*3 (R&D Systems; Minneapolis, MN), 10^−7^ M dexamethasone (Sigma-Aldrich), 50 *μ*g/mL ascorbate-2-phosphate, 40 *μ*g/mL proline, 100 *μ*g/mL pyruvate, and 50 mg/mL ITS+Premix (Becton Dickinson)). For microscopy, the pellets were embedded in paraffin, cut into 4 *μ*m sections, and stained with Toluidine Blue [16].

#### 2.5.4. Immunohistochemistry

Four-micrometer-thick sections obtained from the tissue specimens were deparaffinized and incubated with primary anti-human collagen II antibody (ab34712, Abcam, Cambridge, UK) for 30 min at 4°C. The sections were rinsed again with PBS repeatedly and then incubated with immune-peroxidase polymer secondary antibody (Histofine, Nichirei Biosciences, Tokyo, Japan) for 30 min. Antibodies were visualized by treating the sections with diaminobenzidine tetrahydrochloride (ChemMate DAB+ Chromogen, DakoCytomation) for 5 min. To assess the specificity of immunohistochemistry of COLII, the sections of human chondrosarcoma were stained as described above as our positive control.

#### 2.5.5. Epitope Profile

One million passage-3 cells were resuspended in 200 *μ*L of PBS containing a fluorescein isothiocyanate- (FITC-) or phycoerythrin- (PE-) coupled antibody. The FITC- or PE-coupled antibodies against CD34, CD44, CD45, CD90, CD147, and CD271 were from BD Biosciences (San Diego, CA), CD117 was from eBioscience (San Diego, CA), and CD105 and CD166 were from Ancell (Bayport, MN). Vascular endothelial growth factor receptor 2 (VEGFR2) was obtained from R&D Systems (Minneapolis, MN). Cell fluorescence was evaluated by flow cytometry using an EPICS XL instrument (Coulter Co., Tokyo, Japan), and data were analyzed using a MultiCycle AV analysis program (Phoenix Flow System, Inc., San Diego, CA) [20].

### 2.6. Quantitative Real-Time RT-PCR

Cells that entered adipogenesis or osteogenesis were trypsinized, and total RNA was obtained with RNeasy mini plus kit (Qiagen) according to the manufacturer's protocol. The chondrogenic pellet was homogenized using BioMasher (Nippi, Tokyo, Japan), and total RNA was prepared from a single pellet using RNeasy micro plus kit (Qiagen, Hilden, Germany). After total RNA was reverse-transcribed, cDNA was assayed three times with 10 TaqMan probes for adipogenesis, osteogenesis, and chondrogenesis. For adipogenesis, adipsin (Complement Factor D; Hs00157263_m1), peroxisome proliferator-activated receptor *γ* (PPARG; Hs01115513_m1), and lipoprotein lipase (LPL; Hs00173425_m1) were used. For osteogenesis, collagen type I alpha 1 (COL1A1; Hs00164004_m1), alkaline phosphatase placental type (ALP; Hs03046558_s1), runt-related transcription factor 2 (RUNX2; Hs00231692_m1), and bone gamma-carboxyglutamate protein (BGLAP; Hs01587814_g1) were used. For chondrogenesis, collagen type II alpha 1 (COL2A1; Hs00264051_m1), collagen type X alpha 1 (COL10A1; Hs00166657_m1), and sex determining region Y-box 9 (SOX9; Hs00165814_m1) probes (Applied Biosystems) were used. To detect an adequate endogenous control gene, we performed gene expression assay with TaqMan Array Human Endogenous Control (Applied Biosystems) using s001658 (ACTB; Hs99999903_m1) as a relevant control for normalization of gene expression (data not shown). StepOnePlus™ Real-Time RT-PCR System was used for the quantification and analysis was performed using StepOnePlus™ software (version 2.0, Applied Biosystems).

### 2.7. Statistical Analysis

To assess the difference in isolation status and the character of cells from the synovium of knee and hip, we used Mann–Whitney *U* test. Statistical analyses were performed using SPSS (version 13, SPSS Inc., Chicago, IL) software package. The level of significance was set at a probability value of <0.05.

## 3. Results

### 3.1. Macroscopic and Histological Features of Synovium

On macroscopic analysis, the knee synovium was yellowish and partially floated in phosphate-buffered saline. On the other hand, the hip synovium appears to be white and sank to the bottom as shown in Figures 2(b) and 2(c). Sections from both synovial samples stained with HE are shown in Figures 2(d) and 2(e). Synovium from knee joints consisted of both fibrous tissues and fatty tissues whereas synovium from hip joints appeared to have more fibrous tissues than knee joints.

### 3.2. Cell Isolation from Tissue

**Figure 1:**
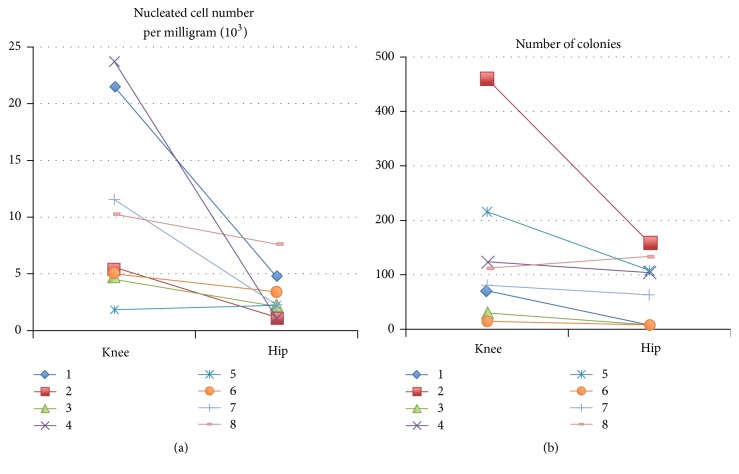
Data on cell samples obtained from the synovium of each case on 8 donors.

Mean sample weight and nucleated cell number per milligram are shown in Table 2. The nucleated cell number per milligram of the synovium from knee was significantly higher than that of the hip (knee: 10.5 ± 8.1 × 10^3^/mg versus hip: 3.1 ± 2.2 × 10^3^/mg, *P* = 0.02). Data on the nucleated cell number per milligram and number of colonies of each case is shown on Figure 1. The nucleated cell number per milligram of the synovium and CFU from the females were higher than males (nucleated cell number per milligram, knee: 12.9 ± 8.0 in female versus 3.2 ± 1.9 in male, hip: 3.4 ± 2.5 in female versus 2.2 ± 0.1 in male) (CFU knee: 143 ± 160 in female versus 123.0 ± 130 in male, hip: 79.2 ± 63.8 in female versus 59.0 ± 69.3 in male). However, it was difficult to assess whether there were significant differences because only two male patients were included in the study.

**Figure 2:**
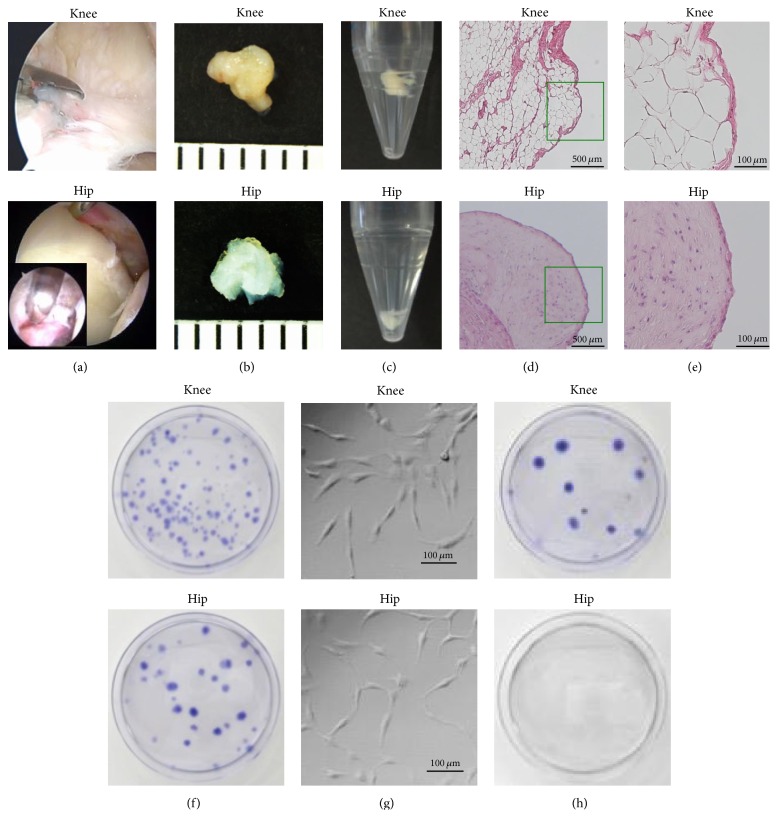
(a) Arthroscopic observation and sample harvesting. (b), (c) Macroscopic features of from knee and hip synovium were examined on a 1 mm scare and in PBS. (d), (e) Histological analysis was performed on tissues stained with hematoxylin and eosin (HE). (f) Representative cell colonies derived from synovium of a donor stained with crystal violet showing large colonies when the nucleated cells were plated at 10^4^ cells/dish. There was no significant difference in the number of colonies between both MSCs from knee and hip joints. (g) Representative morphologic features of the cells magnified at 14 days (passage 0) showing fibroblastic spindle cell morphology. Bar = 100 *μ*m. (h) Representative cell colonies stained with crystal violet derived from the synovial fluid of a donor showing large colonies from the knee, but no colonies from the hip.

In order to gain a maximum number of colonies per amount of nucleated cells, we examined the effect of plating density on nucleated cells from each type of mesenchymal tissue from the 8 donors. From synovium of the knee and the hip, large single cell-derived colonies occurred when nucleated cells were plated at 10^4^ cells/60 cm^2^ dish. The optimal initial cell density was determined as 10^4^/60 cm^2^ dish (Figure 2(f)). There was no significant difference in the number of colony-forming units per 10^4^nucleated cells between knee and hip (*P* = 0.33) (Figure 1 and Table 2). The cells from both types of synovium seemed to be similar in fibroblastic cell morphology (Figure 2(g)).

There was no significant difference of CFU-f regarding gender difference in each of the joints.

### 3.3. Cell Isolation from Synovial Fluid

Sample volume and nucleated cell number per milligram of each synovial fluid in both the knee and the hip joints are shown in Table 3. There was no significant difference of nucleated cell number per milligram of the synovial fluid between the knee and the hip (knee: 5.7 ± 10.5 × 10^5^/mL versus hip: 3.2 ± 7.5 × 10^5^/mL, *P* = 0.20). Colonies were observed in all samples obtained from the knee synovial fluid but not in all samples from the hip synovial fluid (Table 3 and Figure 2(h)). Thus, analysis to compare number of colonies between the synovial fluids of the knee and the hip was not performed. The number of colony-forming fibroblasts was 3.3 ± 4.9 in the knee joints.

### 3.4. In Vitro Expandability and Cell Viability Assay

**Figure 3:**
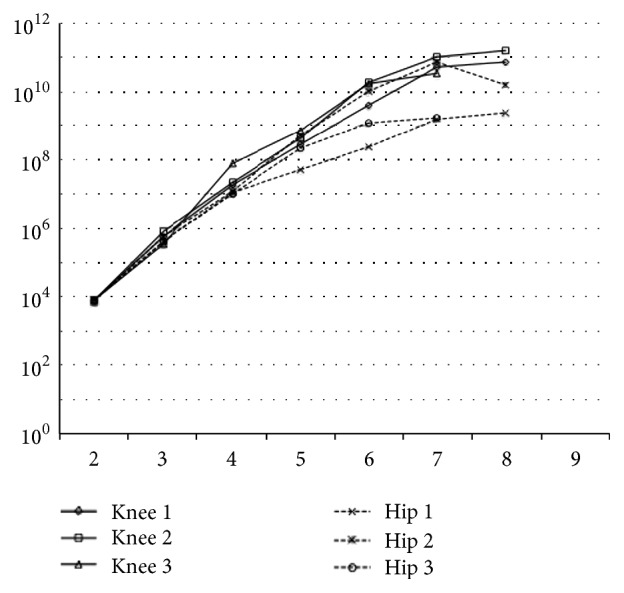
Proliferating potential of both hip and knee cells on 3 donors. Both passage-1 cells were plated at 50 cells/cm^2^ on 150 cm^2^ dishes. Thereafter, the cells were replated at 50 cells/cm^2^ every 14 days until their expansion potential was lost. The expandabilities were lost at passage 8 in both MSCs derived from knee and hip joints.

**Figure 4:**
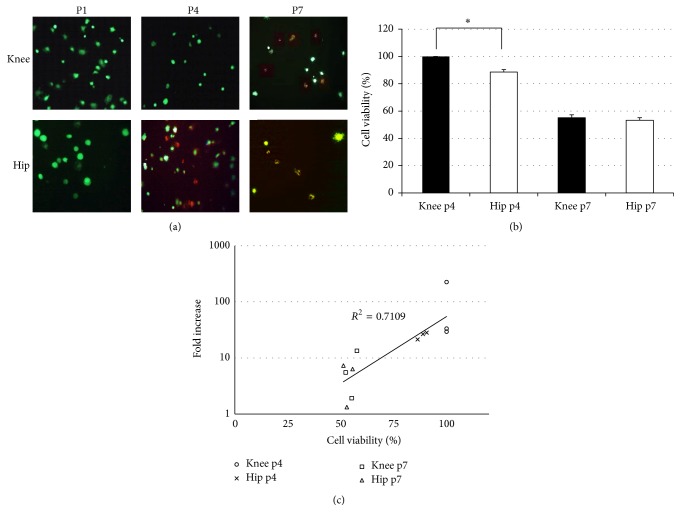
(a) LIVE/DEAD Viability/Cytotoxicity Kit assay. At passage 1, green fluorescence was observed in all cells derived from both joints, confirming that these cells were alive. Passage-4 cells derived from knee were alive, but the part of cells from hip was dead. At passage 7, the half of both cells was dead. (b) Cell Counting Kit-8 assay for passages 4 and 7 cells on 3 donors. The bar means standard deviation. At passage 4, viability of cells derived from knee was significantly higher than those from hip (*P* < 0.01, Mann–Whitney *U* test). At passage 7, there was no significant difference of cell viability between knee and hip. ^**∗**^Significant difference. (c) The correlation between the fold increase and cell viability on each sample at passages 4 and 7.

Expandability was lost after eight passages for both types of synovium-derived cells (Figure 3). Using a LIVE/DEAD Viability/Cytotoxicity Kit, both types of passages 1 to 3 cells were determined to be alive. In passage 4, cells derived from the knee synovium were thriving but cells derived from the hip synovium were losing viability (Figure 4(a)). Using a Cell Counting Kit-8 assay, viability of cells derived from the knee synovium was significantly higher than that of cells derived from the hip synovium (knee versus hip, 100% versus 88.4%, *P* < 0.01, Mann–Whitney* U* test) (Figure 4(b)). On the other hand, the half of both cells was dead at passage 7. At passage 7, there was no significant difference of cell viability between knee and hip with both cell lines nonviable. In addition, there was a strong correlation between the fold increase and cell viability (*R*^2^ = 0.71) (Figure 4(c)).

### 3.5. Yield Obtained

The live passage-0 cell number per colony of synovium from knee and hip was 2.3 ± 1.4 and 3.8 ± 2.8, respectively (×10^5^ cells/mg, *P* = 0.23, Mann–Whitney *U* test). There was no significant difference in the yield of live cells obtained from both samples (knee versus hips, 15.5 ± 22.2 × 10^6^ versus 5.2 ± 4.9 × 10^6^ cells, *P* = 0.16) (Table 2).

### 3.6. Adipogenesis

**Figure 5:**
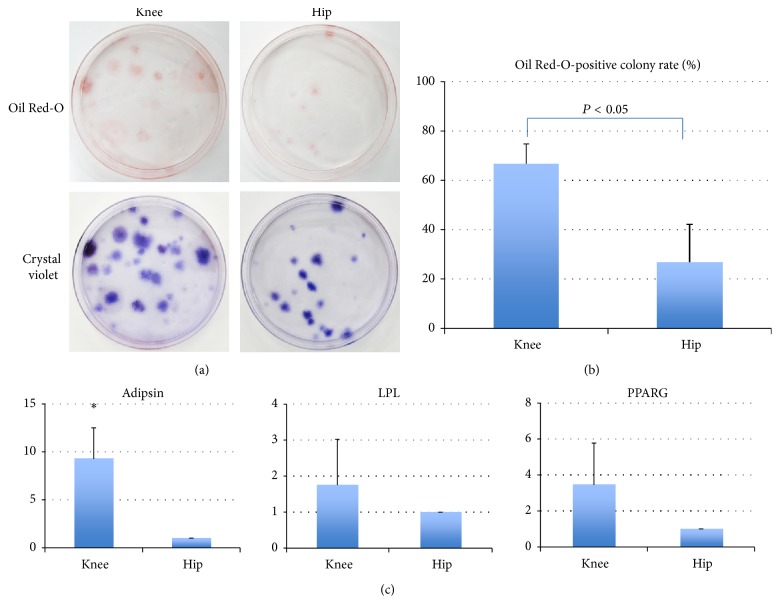
Adipogenic potential about each synovial cell. (a) Colonies staining positively with Oil Red-O and crystal violet in each cell. (b) Proportion of Oil Red-O-positive colonies in relation to the total number of colonies in each MSC. Values are shown as the mean and standard deviation of 6 donors. ^**∗**^The rate of Oil Red-O-positive colonies from the knee was significantly higher than that from hip (Mann–Whitney* U* test, *P* < 0.0001). (c) Quantitative real-time PCR data from 3 representative donors are shown. The bar means standard deviation.

In an adipogenesis differentiation assay, all samples generated colonies of adipocytes, which stained with Oil Red-O (Figure 5(a)). However, the rate of Oil Red-O-positive colonies was significantly higher in cells derived from the knee as compared to those from the hip (Figure 5(b); *P* < 0.05, Mann–Whitney* U* test). In addition, real-time quantitative PCR analysis for adipogenic gene expression was performed (*n* = 3). As shown in Figure 5(c), the gene expression of* adipsin* in the knee group was significantly higher than those in the hip (*P* < 0.05, Mann–Whitney *U* test). There were no significant differences in gene expression of PPARG and LPL between both joints (*P* = 0.26 and 0.26, Mann–Whitney *U* test).

### 3.7. Osteogenesis

**Figure 6:**
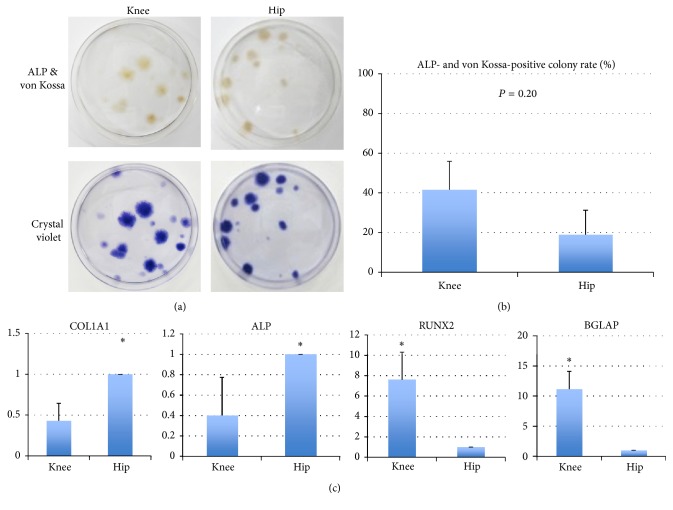
Osteogenic potential of each type of MSCs. (a) Colonies stained positively with von Kossa and ALP as well as crystal violet. (b) Proportion of von Kossa and ALP-positive colonies in relation to the total number of colonies. Values are the mean and standard deviation of 6 donors. ^*∗*^There was no significant difference in the rate of von Kossa and ALP-positive colonies between knee and hip (Mann–Whitney* U* test, *P* = 0.20). (c) Real-time RT-PCR. Gene expressions of* COL1* and* ALP* in knee were significantly lower than those in hip (Mann–Whitney *U* test, *P* < 0.05); on the other hand gene expressions of* RUNX2* and* BGLAP* in the knee were significantly higher than in the hip (Mann–Whitney *U* test, *P* < 0.05). The bar means standard deviation.

In an osteogenesis differentiation assay, cells derived from the knee synovium generated colonies of osteogenic cells that stained with von Kossa/ALP double staining (Figure 6(a)). Rates of von Kossa- and ALP-positive colonies in relation to the total number of colonies between cells derived from the knee and the hip joints were found to be not statistically different between the knee and the hip (Figure 6(b)) (*P* = 0.20, Mann–Whitney* U* test). According to real-time PCR assay, the levels of gene expressions of* COL1* and* ALP* in cells from the hip were significantly higher than those in cells from the knee (3 donors, Figure 6(c); *P* < 0.01 (COL1A1), *P* = 0.04 (ALP)). In contrast, the gene expressions of* RUNX2* and* BGLAP* genes in cells from the knee were significantly higher than those in cells from the hip (3 donors, Figure 6(c); *P* < 0.01 (RUNX2), *P* < 0.01 (BGLAP), Mann–Whitney *U* test). These findings indicate that osteogenic potential of MSCs from the hip joint is equivalent to that from the knee joint, but osteogenic rate of MSCs from the knee joint is greater than that from the hip joint [21].

### 3.8. Chondrogenesis

**Figure 7:**
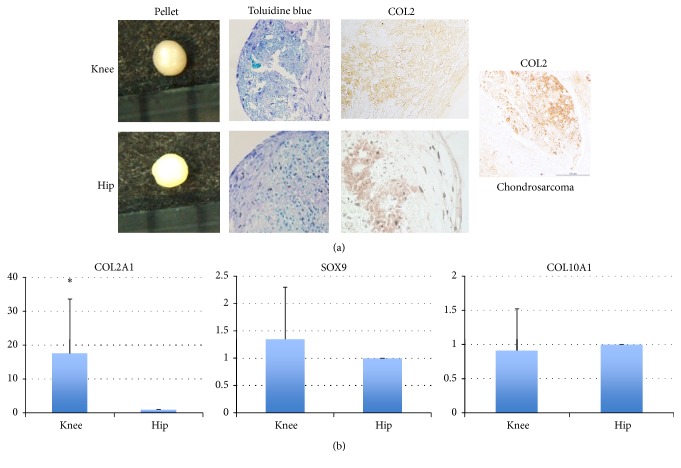
Chondrogenic potential of each type of synovial cell. (a) The pellets from each sample were shown. There was no significant difference in the diameter of pellets between knee and hip (*P* = 0.82, Mann–Whitney *U* test). Histological analysis was performed on tissues stained with Toluidine Blue staining and immunocytochemical analysis with type II collagen by tissue source and human chondrosarcoma for positive control. (b) Real-time RT-PCR. The bar means standard deviation. Expression of* COL2* gene in knee was significantly higher than that in hip (*P* < 0.05, Mann–Whitney* U* test). ^**∗**^Significant difference.

In chondrogenesis differentiation assays, most of the pellets from each tissue were >0.5 mm in diameter. The pellets of both samples had positive Toluidine Blue stained cartilage matrix. There was no significant difference of the mean diameter of pellets between both pellets (knee: 825 *μ*m versus hip: 800 *μ*m, *P* = 0.82, Mann–Whitney *U* test). Immunoreactivity for type II was found in both pellets (Figure 7(a)) but immunoreactivity for type X collagen was not. The real-time PCR study demonstrated that the gene expression of* Col2A1* in pellets derived from the knee was significantly higher than that in pellets derived from the hip (Figure 7(b); *P* < 0.01, Mann–Whitney *U* test). There were no significant differences of the expressions of COL10A1 and SOX9 between both pellets (Figure 7(b)). These findings suggest that the chondrogenic potential of MSCs from the hips is nearly comparable to that from the knee joint despite the expression of Col2A1 from the knee MSCs being superior to that from the hip MSCs.

### 3.9. Epitope Profile

**Figure 8:**
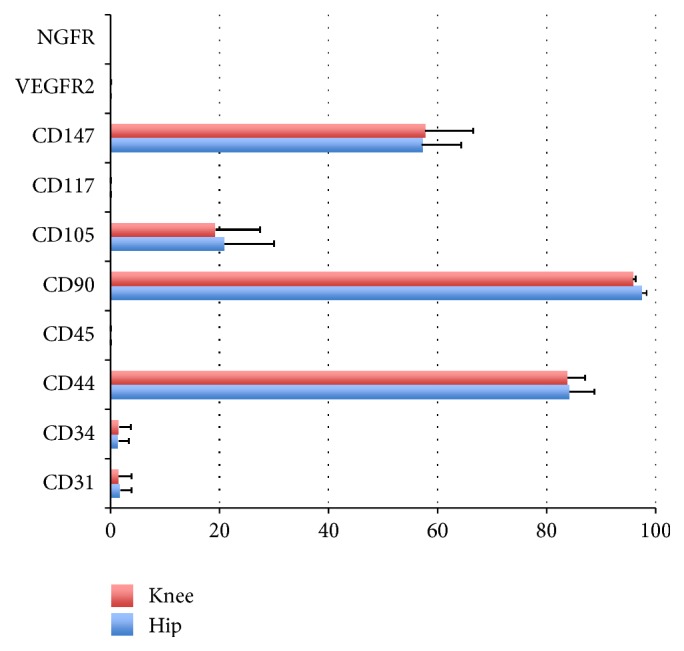
Flow cytometry analysis of the expression of cell surface markers related to stem cells, hematopoietic stem cells, and endothelial cells in each population of cells derived from each sample. Passage-2 cells from 3 donors were examined, and similar results were obtained. Positive expression rates are shown.

The rate of positivity for CD45 (hematopoietic cell marker), CD31 (endothelial cell marker), CD117 (C-kit, stem cell factor receptor), CD34 (hematopoietic progenitor cell antigen), CD166 (ALCAM, SB10), VEGFR2 (Flk-1), and NGFR was <2% in cells derived from both types of synovium (Figure 8). The mean rate of positivity for CD44 (hyaluronan receptor) from cells of the knee and the hip was 83.8% and 84.1%, and CD90 (Thy1) [22] was 95.8% and 97.4%. Similarly, positivity for CD105 (SH-2) from cells of the knee and the hip was 19.3% and 21.0%, respectively, and CD147 (neuregulin) [23] was 57.7% and 57.1%, respectively (Figure 8). These findings showed that surface markers of each of the cells were similarly expressed [19].

## 4. Discussion

This study has four major findings. First, we determined that adherent cells derived from the synovium of the hip joints can be isolated with consistent proliferation and multilineage differentiation potentials. Second, we found that colony-forming units, fibroblastic morphology, and epitope profile of MSCs derived from synovium of the hip joints were similar to MSCs derived from synovium of the knee joints. Third, there was a difference in the number of nucleated cells derived from synovium per milligram, colony-forming units derived from synovial fluid, proliferation rate, adipogenesis, osteogenesis, and the expression of COL2A1 during chondrogenesis in MSCs synovium from the knee joints compared to the hip joints. Fourth, the yield obtained from synovium of the knee joints was slightly higher but not more significant than the hip joints.

One of the strengths of this study is that we compared the proliferation and differentiation potentials between synovial MSCs of two joints from the same donor. Previous studies demonstrated that the condition of the donors could potentially affect the ability of MSCs to function and suggested that MSCs differ in property and vary from donors to donors [24–26]. By providing same donor samples, we are avoiding this potential bias. Siegel et al. demonstrated that age and gender affect the growth potential of MSCs derived from human bone marrow [26]. Lee et al. demonstrated that estradiol affected MSCs' potential and proliferation rate in a porcine animal model [27]. They concluded that MSCs of females were affected by menopause. Matsukura et al. demonstrated that a longer period of time between onset of pain and surgery in patients with meniscus injury increased the amount of MSCs derived from human synovial fluid [24]. Sekiya et al. also demonstrated that MSCs derived from human synovium in patients with severe osteoarthritis have greater potential of proliferation compared to healthy patients [25]. However, they did not find any significant difference when comparing onset of pain to surgery or severity of osteoarthritis (OA).

In this study, we isolated adherent cells derived from synovium of the hip and the knee joints. Our findings demonstrated that these synovial cells from both the knee and the hip joints have proliferation and multilineage differentiation potentials. MSCs from both joints had a morphologically spindle shape, similar to that of fibroblasts, with functional capacities to proliferate and differentiate into multilineage cells. In addition, cells from both joints expressed similarly surface markers. Sakaguchi et al. and Segawa et al. demonstrated that cells derived from human synovium and bone marrow were negative for CD34 and CD45 and positive for CD90, CD105, and CD44 [14, 20]. Similarly, our findings reveal that expressions of both cells were negative for CD34, CD45, CD31, CD117, NGFR, and VEGFR2 and positive for CD44, CD90, CD105, and CD147. These findings indicate that both knee and hip synovial derived MSCs expressed similar cell surface epitopes to those from other cell sources including bone marrow and adipose tissues. International Society for Cellular Therapy position statement defined that MSCs are as follows: (1) adherent cells to plastic, (2) specific surface marker expression, and (3) multipotent differentiation potential. In this study, we were able to define MSCs from both the hip and the knee joints according to the criteria stated above.

In this study, we investigated the yield obtained and expandability as part of the proliferation potential of MSCs derived from synovial fluid and synovial tissue in both the knee and the hip joints. Our data demonstrated that there were no significant differences in colony numbers per 10^4^ nucleated cells, number of nucleated cells per colony between the hip and the knee joints. In addition, the expandability of MSCs from both the knee and the hip was lost at the same time point (passage 8). However, the number of nucleated cells per milligram derived from synovium in the knee was significantly higher than that derived from that in the hip joint. Yield obtained derived from synovium in the knee joints was also not significantly, but still higher than in the hip joints. MSCs derived from synovial fluid of the hip were not capable of proliferating at passage 0. Indeed, LIVE/DEAD Viability experiments determined that both types of passage 1 cells were alive. At passage 4, cells derived from knee synovium were still alive. In contrast, reduction of viability was evident in those MSCs derived from the hip joint. These findings suggest that the potential of proliferation derived from synovial MSCs in the knee joints was superior to those in the hip joint.

Previously, it has been suggested that the potential of synovial MSCs can be affected by the presence of cartilage injury and the period between the onset of pain and date of surgery [28]. However, in this study, there was no significant difference in growth potential despite varied time frame between onset of pain and date of surgery for both the knee and the hip joints for our patients.

We also investigated the multilineage differentiation capability of MSCs derived from synovium in the hip and the knee. Our findings revealed that adipogenic differentiation potential in the knee joint was significantly higher than that in the hip joints. Interestingly, our histological findings revealed that synovial tissues from the knee joints appear to have slightly more fatty tissues than from the hip joints. These findings may be associated with why the hip synovial MSCs have lower adipogenic differentiation potential than the knee synovial MSCs. In fact, Mochizuki et al. described that the fibrous knee synovium, which is distinct from adipose synovium (infra patella fat pad), has comparable chondrogenic potential with adipose synovium and fibrous synovium has lower adipogenic potential than adipose synovium in the knee joint [29]. Our histological finding of the knee synovial tissue appears to be similar to theirs of adipose synovium.

Our findings also revealed that osteogenic differentiation potential in the knee joints was higher than that in the hip joints. The rate of von Kossa- and ALP-positive colony was not significantly different between both joints; however the expressions of RUNX2 and BGLAP in synovial MSCs derived from the knee joints at real-time PCR were significantly higher than the hip joints. On the other hand, the expressions of ALP in synovial MSCs derived from the knee joints at real-time PCR were significantly lower than the hip joints. ALP is an early marker of osteogenesis. Contrarily, RUNX2 is a late marker of osteogenesis [21, 30]. Thus, our findings suggest that synovial MSCs derived from the knee differentiated more rapidly than those from the hip. This study determined that the potential of osteogenic differentiation of synovial MSCs from the knee was higher and faster than that from the hip.

Previous studies addressed the potential of the knee synovial MSCs for expansion and multiple mesenchymal lineage differentiation [11, 12]. De Bari et al. first described that synovium membrane derived cells had extensive expansion potential in monolayer culture [11]. Sakaguchi et al. demonstrated that synovium from the knee was superior as a potential source of MSCs among five tissues (bone marrow, synovium, periosteum, skeletal muscle, and adipose tissue). They noted that knee synovial MSCs had the greatest expansion and differentiation ability among these tissues [12]. In addition, our previous study revealed that the shoulder synovial MSCs derived from subacromial bursa which were retained at passage 10 had the greatest differentiation potential compared to other tissues such as bone marrow, entheses, and bone [19]. In keeping with these studies, we compared potential of MSCs derived from synovial tissues in the knee and the hip joints within the same donor. Our findings indicated that synovial MSCs from the knee joints have superior capability of giving rise to adipogenic and osteogenic tissues compared to those from the hip joints. In addition, our results suggest that synovial MSCs from the knee have a higher yield compared to the hip.

In this study, we demonstrated that synovial MSCs from the knee have greater potential and differentiation capacity to those in the hip in the same donors. We showed that the nucleated cell number per milligram of sample from the knee synovium was significantly higher than that from the hip synovium. The reason why MSCs in the hip have a lower potential of MSCs than that in the knee remains unclear.

*Study Limitations*. There are some limitations in this study. First, our study population was small. The prevalence of simultaneous hip and knee arthroscopy is very low in the normal practice. Thus, it is extremely difficult to get the opportunity to collect synovial tissues from both joints on the same day. There is the risk of sample bias with proliferation assay due to the small sample size. Second, since this is an in vitro study, we are unable to demonstrate the potential of these MSCs in vivo. Third, we did not compare synovial MSCs to MSCs from other sources including bone marrow or adipose tissues. Fourth, histological characteristic of the knee synovium was different from the hip synovial tissue in this study. In order to eliminate the possibility of contamination of fat pad tissue or joint capsule, we obtained the tissue sample consistently by harvesting the synovium near the torn meniscus or acetabular torn labrum. Fifth, in real-time PCR study, there is possibility that interdonor variability could be larger than the difference between the knee and the hip joint.

In conclusion, our findings suggest that isolated MSCs from the hip synovial cells have self-renewal and multilineage differentiation potentials. However, adipogenesis and osteogenesis potentials of MSCs from the knees are superior to those of MSCs from the hips in the same donor. MSCs derived from the knee synovial tissue may be a better source for future mesenchymal stem cell therapy.

## Figures and Tables

**Table 1:** Patient demography, osteoarthritis grading, and arthroscopic findings of hip and knee joints.

Number	Age	Sex	BMI	Knee						Hip							
				Period from onset to surgery (months)	X-ray Kellgren-Lawrence grade	Side of meniscus tear	Condition of meniscus	Cartilage ICRS grade		Period from onset to surgery (months)	X-ray Kellgren-Lawrence grade	Position of labral tear	Labral condition	Cartilage ICRS grade and zone			
														Acetabulum		Femur	
								Femur	Tibia								
														Grade	Zone	Grade	Zone
1	25	F	22.6	36	0	LM	Degenerative	0	2	120	0	12:00–2:00	Normal	0	—	3	3
2	44	F	20.8	240	2	LM	Degenerative	4	4	24	0	2:00–10:00	Degenerative	1	4	1	3
3	61	M	23.3	8	1	LM	Normal	0	0	8	0	11:00–2:00	Normal	0	—	3	3
4	64	F	25.7	108	2	MM	Degenerative	3	3	22	2	9:30–3:00	Degenerative	4	3	4	3
5	30	M	26.1	1	0	LM	Normal	0	0	11	1	1:30–2:00	Normal	0	—	0	—
6	51	F	21.2	2	1	LM	Normal	0	0	4	1	11:00–12:30	Degenerative	0	—	0	—
7	64	F	23.8	11	1	MM	Degenerative	3	0	8	0	12:00–2:00	Degenerative	0	—	0	—
8	44	F	20.5	84	0	LM	Normal	2	0	132	0	12:00–2:00	Normal	3	3	0	—

LM: lateral meniscus; MM: medial meniscus.

**Table 2:** Data on cell samples obtained from the synovium of 8 donors. Values are shown as mean ± standard deviation. Nucleated cells were plated at 10^4^/60 cm^2^.

Source	Sample weight (mg)	Number of nucleated cells (×10^3^)/mg	Number of colonies	Number of nucleated cells/colony	Yield obtained (×10^6^)
Knee	53.2 ± 31.3	10.5 ± 8.1^*∗*^	138.5 ± 144.1	2.3 ± 1.4	15.5 ± 22.2
Hip	85.3 ± 65.3	3.1 ± 2.2	74.2 ± 60.7	3.8 ± 2.8	5.2 ± 4.9

^*∗*^ *P* < 0.05; Mann–Whitney *U* test. The number of nucleated cells per sample weight derived from knee was significantly higher than from hip.

**Table 3:** Data on the cell samples derived from synovial fluid of 8 donors. Values are shown as mean ± standard deviation.

Source	Sample volume (ml)	Number of nucleated cells (×10^5^)/ml	Number of colonies	Number of nucleated cells/colony	Yield obtained (×10^5^)
Knee	1.0 ± 0.0	5.7 ± 10.5	3.3 ± 4.9	6.7 ± 11.7	1.6 ± 3.1
Hip	1.2 ± 0.4	3.2 ± 7.5	0	0	0
